# The adult and pediatric palliative care: differences and shared issues

**DOI:** 10.1186/s44158-023-00085-8

**Published:** 2023-01-12

**Authors:** Cosimo Chelazzi, Gianluca Villa, Iacopo Lanini, Stefano Romagnoli, Nicola Latronico

**Affiliations:** 1 Department of Medical and Surgical Specialties, Radiological Sciences and Public Health, University of Brescia, Brescia, Italy; 2 Unit of Palliative Care and Integrated Home Service, Spedali Civili University Hospital, Brescia, Italy; 3 Department of Health Sciences, Section of Anesthesia, Intensive Care and Pain Medicine, University of Florence, Florence, Italy; 4 Department of Anesthesia and Intensive Care, Section of Oncological Anesthesia and Intensive Care, Azienda Ospedaliero Universitaria Careggi, Florence, Italy; 5 Fondazione Italiana Di Leniterapia, Florence, Italy; 6 Department of Anesthesia, Critical Care and Emergency, Spedali Civili University Hospital, Brescia, Italy; 7 “Alessandra BONO” University Research Center On LOng Term Outcome (LOTO) in Survivors of Critical Illness, University of Brescia, Brescia, Italy

**Keywords:** Palliative care, Service, Organization, Adult, Pediatric

## Abstract

Adult and pediatric palliative care (PC) share common aims and ethical principles but differ in many organizational and practical aspects. The aim of this narrative review is to analyze these differences and focus on which key aspects of pediatric palliative care could integrate adult services for a better care of suffering patients.

Interventions which are peculiar of pediatric PC respect to adult PC include: an earlier referral to the PC service to identify the needs and plan the interventions at an earlier stage of the disease; consequently, a more systematic cooperation with the disease-specific physicians to reduce the burden of treatments; a better integration with the community and the social surroundings of the patients, to prevent social isolation and preserve their social role; a more dynamic organization of the PC services, to give patients the chance of being stabilized at in-hospital or residential settings and subsequently discharged and cared at home whenever possible and desired; the implementation of respite care for adults, to help the families coping with the burden of the disease of their beloved and promote the home-based PC.

This review underlines the relevance of some key-aspects of pediatric PC that can be beneficial also within PC of adults. Its findings give the chance for a more dynamic and modern organization of adult PC services and may serve as a basis of future research for new interventions.

## Background

Palliative care (PC) is both a medical specialty and a way of providing medicine and care [1]. Every time we focus not only on “curative” or etiologic treatments, but also on treating the symptoms and the burden of disease, i.e., relieving the suffering, we are actually practicing “palliative care”. In its broader, primary meaning, PC is not only the provision of sedation and analgesia at the end of life (i.e., “hospice care”), but also of relief from disease-related anxiety, secondary effects of treatments and, finally, psychosocial, spiritual, personal, and financial issues raised by the loss of health related to a life-threatening disease [2, 3]. If modern definition of “health” relies on self-perception of oneself healthy state, PC should aim at restitution of this perception to the “ill” person. Aims of PC are thus not predetermined or limited to objective outcomes (e.g., 90-day hospital survival or ventilator-free days), but they are more related to the single person’s health perception and needs [2]. Eliminating pain and emotional, psychological, social, and spiritual suffering is the aim of palliative care and helping some patients to cope with their life-threatening disease may require more complex interventions in other domains than pain and anxiety. PC emphasizes the fact that it is the whole person and not just its body who suffers, and that suffering extends beyond the physical domain and may exist even in a person devoid of symptoms [4]. There can be no effective care of suffering without recognizing the whole person. This review focuses on similarities and differences between adult and pediatric PC, looking at how they can share some issues and methods of solving them starting from a broader vision of PC.

### The relevance of the problem

The World Health Organization (WHO) defined PC as “the prevention and relief of sufferings of the adult and pediatric patients and families facing the problems associated with life-threatening illness” [5]. Palliative care can be devoted to the care of adults (adult palliative care, APC) or to children and young adults (pediatric palliative care, PPC). Both APC and PPC are widely recognized as an essential components of modern health care that national systems should endorse and provide to their people. Traditionally, patients referred to APC are those with life-threatening diseases, such as end-stage cancer or terminal cardiac/respiratory diseases or advanced dementias. As for the children, more conditions potentially fall under the interest of PPC practitioner. The Royal College of Pediatrics and Child Care proposed a classification of life-threatening diseases that may need PPC services at some point during their course [6] (see Table 1). This classification may comprise different diseases in countries with different incomes. Indeed, while in high-income countries (HIC), cancer and congenital/neuromuscular diseases are most prevalent, in low-/middle-income countries (LMIC), children with HIV sequelae, multi-drug-resistant tuberculosis and famine, as well as cancer, may need PPC. Of note, while most of children who need PPC come from the LMICs, PC services are rarely available in these areas.

**Table 1:** Royal College of Pediatrics and Child Care’s classification of life-threatening diseases (Bogetz et al., 2014; Mellor and Hain, 2010)

Description of disease	Examples
Conditions that can be treated but retain high possibility of death	Acute leukemia; cancer
Progressive conditions that lead to death but have long-term treatments, including intensive care	Down syndrome; muscular dystrophy; cystic fibrosis
Conditions without specific treatment but requiring palliative care; ultimately terminal	Severe congenital/metabolic disease
Severe neurologic impairments in which complications may lead to early death	Severe cerebral palsy

Such a wide definition of PC makes the numbers for potential APC/PPC patients very large [7]. The WHO estimates that about 40 million people need PC yearly in the world; roughly, just 14% of those people will receive PC. For children, WHO estimates that 21 million people < 20 years old may need PC each year with a death toll that can reach up to 2.5 million/year. European regional data are more difficult to obtain. In a retrospective survey on adult PC services in Europe, Pivodic et al. found that in the years 2009–2010, general practitioners’ reported deaths were 4466 in Belgium, Spain, The Netherlands, and Italy; interestingly just 29% of patients in the Netherlands, 39% in Italy, 45% in Spain, and 47% in Belgium (*p* < 0.001) were provided with specialistic PC [8]. This unmet need for PC is even larger in LMIC, where only a fraction of patients will receive PC [9]. These unmet needs have been considered by the WHO and the European Union, among others, as no longer tolerable and PC is now seen both as a right of the individual and an indicator of good quality healthcare. Providing palliative care should be seen as a principle of social, economic, and political justice, other than a basic humanitarian principle and improving education on these issues is pivotal to achieve good quality of care [10, 11].

### What adult and palliative care do share

The aims of APC and PPC are the same. As underlined above, both focus on control of symptoms and relief of pain and other types of suffering rather than only on curative treatments. However, PC is not mutually exclusive with curative treatments. On the opposite, PC can be concomitant to other, disease-specific treatments, such as chemotherapy, thus increasing compliance of patients to uncomfortable therapies and potentially contributing to a better outcome. PC has been endorsed as a mean to provide more comfortable care by several organizations, including WHO and European Union [12]. The implementation of PC and organization of PC services have been seen as indicators of good quality healthcare. As noted above, a great uneven availability of PC services and access to PC drugs is observed between high-income countries and LMIC, where those services would be needed the most [9].

Both APC and PPC are underpinned by the same bioethical principles [13]. Bioethics and PC shared a common evolution through the history of modern medicine and are partially overlapping as to their role in clinical decision-making. Depending on hospitals policy or organizations, bioethicists can be sometimes consulted to contribute an opinion in difficult clinical situation (e.g., “should this treatment be withdrawn?”). On the opposite, PC specialists may sometimes provide bioethical consult in difficult clinical scenarios, even without been actively involved in clinical management [13]. The four common ethical principles of autonomy, beneficence, non-maleficence, and justice are the founding principles of any clinical practice, including PC. There is hierarchy, since it has been an evolution from a paternalistic approach, where the beneficence was prevalent on patients’ autonomy, to shared clinical decision making, with autonomy being the leading principle, underlying the importance of patients’ self-determination [14, 15]. This implies that either disease specific treatments and palliative treatments need to be in line with the single patient’s needs and aims, independently from how positive the effect would be in doctors’ mind. Tightly linked to the principle of autonomy is the need of informed consent that should be obtained also for PC clinical practice, as part of an open and honest curative relationship. Since “suffering is, ultimately, a personal matter” [4], PC practice gives the patient the chance of focus on personal aims and priorities and actively participate to the decision-making, clinical process [16]. Personal meanings, beliefs, and values should then be sought and noted by the PC practitioner, considering that not only pain or physical symptoms may contribute to the suffering of patients, but also less “measurable” aspects such as uncertainty about the future and loss of social, professional, or family roles.

PC can be seen to provide more compassionate care to people suffering from any disease and, as such, can be practiced by all healthcare providers, independently by their role or specialization, whenever they alleviate symptoms during their main practice (i.e., “primary palliative care”) [17]. “Secondary” palliative care refers to the care of symptoms or effects of therapies that can be implemented by specialists other than in PC when they take care of their patients (e.g., oncologists treating chemo-therapy related nausea or surgeons prescribing pain killers). “Tertiary” palliative care refers to the activity of PC specialists in scenarios requiring more complex care for suffering relief, not limited to pain and anxiety. It also refers to the activity of organizing PC services and networks, including in-hospital, residential, out-patients, home, and hospice care. Tertiary PC should be integrated in the usual clinical practice, so as to warrant an early referral of patients with cumbersome symptoms or secondary effects of therapies, possibly preventing them before these arise [2].

### What are the differences between APC and PPC?

Despite sharing general aims and ethical principles, APC and PPC are different in many aspects. In general, while the general aim would be to improve quality of life (QOL), the way it is achieved may differ much. Often, QOL for adults can be improved by cessation of cumbersome and futile disease-specific or invasive treatment and focusing on relief of suffering in its many domains. For children, QOL is much more complex concept, and termination of disease-specific treatment is not necessary part of it (see below).

The first and most important point is that while APC is patient-centered and relatives are involved mostly as “surrogates” decision-makers, PPC is “child-and-family” centered, the family being active subject and object of the PC practice [11, 18, 19]. This is true for some reasons: first, the principle of autonomy applies in different ways to adults and children. The adult patient is in general thought to be capable of self-determination and able to decide if the proposed treatments are in line with his needs or expectations. If the patient is unable to decide (e.g., he is unconscious), an advanced plan of care or a living will may be available to help decision makers; a “surrogate” or health attorney may help clinicians to decide [20]. Depending on their age, children may be completely dependent on the decisions of the family or can be mature enough to participate to decision. If this is the case, even though the parents bring the responsibility for the informed consent, children need to be involved in clinical decisions, proportionately to their perceived capability of understanding [6]. In case they express their opinion, parents and doctors would need to listen to them and try to take into consideration what is expressed by the child.

Another key difference is the time of referral to PC services. For the adult patient, this usually occurs at a relatively late stage of a life-threatening disease and in some countries, the APC specialist is still consulted only for the dying patients, typically when the life-span is less than 6 months and/or when all “curative” treatment has been withdrawn or refused by the patient [21]. However, the concept of “simultaneous palliative care” is gaining acceptance in medical oncology (see below) and there is not necessarily a complete “hand-over” from the disease specific specialist to the PC practitioner. For children the referral occurs often much earlier, since life-threatening diseases can start very early, and the families need to be involved early in PC treatments. Thus, PPC easily coexists with active treatments, and the pediatricians or other specialists are frequently involved. Some pediatric conditions are also rare, and specialized expertise is important to manage complex symptoms and define prognosis. Moreover, families of children usually need to know that active treatments are put in place concomitantly to palliation of symptoms, since the emotional burden can be cumbersome [6, 18].

The role of family in PPC goes beyond providing informed consent. Families are usually actively involved in provision of PPC. Since keeping children at home in their comfortable environment is important in PPC, houses need sometimes to be adapted to the progressive loss of physical autonomy of children. Family caregivers need training on the use of physical auxilia or machines such as home ventilators or infusion pumps. Thus, family members can be progressively and actively involved in the care of their ill child. Families are also object of PPC practice: they will often need psychological support for emotional burden linked to the experience of having a child with a life-threatening or invalidating disease and to the tiring care that this implies. Institutional or financial support may be required for this or even to help caring for other, unaffected children they may have. Indeed, life-threatening disease in children affect the family as a whole, and this should be taken care of by the PPC practitioner. The quality of family-driven care will reflect on the residual health of the affected child, so those issues are all of interest for the PPC service which coordinates the process.

The same coordination activity should be implemented with schools, where the child may have special needs [18]. Indeed, due to the long and progressive course of many life-limiting diseases in children, many of them will attend classes at different ages. Learning and social integration should be sought as necessary, since any child, even if ill, has the need and the right to share the same activities of her/his coetaneous, whenever possible. This facilitation of social and scholastic activities is part of the network that a modern PPC service should bring to patients and families, i.e., “legacy” [22].

When symptoms are worsening, clinical PC can be provided in residential or home settings [23]. Residential scenarios are those of in-hospital or hospice care. In PPC, hospice care is dedicated not only to the dying child, but also to those children whose symptoms are worsening, for temporary treatment and stabilization, with the aim of discharging them at home [24, 25]. Home services may include an outreach PC team which attends the child at home, or locally organized, “peripheral” PC [26]. The PPC practitioner should coordinate this activity, tailoring the best path on a case-to-case basis [18]. Sometimes the needs will change over time, and reassessment is essential to always provide the more appropriate organizational and clinical answer.

Some of pediatric life-threatening conditions will transition in the young adult and adult age [27, 28]. Thus, some structured hand-over or planning of care should be foreseen to avoid therapeutic lags. Usually, for very complex syndromes, pediatric referral can be still considered even later in the course of the disease. With transition to the adult age, autonomy of the patient may grow more mature, and this may imply different therapeutic strategies. This transition would need to be built progressively and adapted to the physical/cognitive impairment brought by the disease.

Finally, the respite care refers to a care which temporarily relieves the burden of caregiving from families [29]. Respite care can be provided either at home or in residential scenarios. Providers can be hospital or community-based doctors, nurses, or therapists who will take care of the child for a limited time, relieving the effort from the shoulders of the parents. This can achieve a double aim. First, it gives to families a chance to “pause” from highly emotionally demanding care, allowing them to dedicate time to other issues (e.g., working) or unaffected children [30]. Second, it is a chance to train the parents to use some of the auxilia that may be needed, such as home mechanical ventilators or suctioning tools. Allowing dying children to be cared at home, respite care can contribute to increase the quality of life of children with terminal illnesses and their families [31].

How the pediatric palliative care can inspire the implementation of an adult service.

A modern approach to APC is needed [32] (see Table 2). Hospice care has been defined as the care provided in the last six months of life, when specific disease treatment has been withdrawn (see above) [21]. In some cases, PC can be provided to the critically ill, dying patients [33–35]. However, palliative care can go well beyond this traditional view. It may widen to encompass the concept of global relief from suffering, not only treating the unbearable symptoms of life-threatening diseases, but also taking care of psychological, spiritual, familiar, or even financial issues linked to a lack of the self-perceived sense of being healthy [4]. PC is not pain medicine, and the concept that persons are not just their bodies or their minds but also their personality, families, and social/working backgrounds, is central for a modern approach to PC. More so, bodily symptoms may be unpaired from personal ill-being and treating them may be not sufficient to patients’ relief.

**Table 2:** Interventions which are typical of PPC that can implemented within APC service

Intervention	Aim
·**Earlier referral to PC service**	·Early detection of needs and planning of appropriate therapies
·**Simultaneity with disease-specific treatment**	·Reassuring the patient on continuity of care ·Better coping with cumbersome/painful but potentially curative treatments ·Better definition of prognosis in complicated cases ·Some specific treatments may retain a palliative role in late stages (e.g. radiotherapy)
·**Integration of PC service with community (e.g.**, **schools, workplace, religious community)**	·Prevention of social isolation ·Preserving social, working or family role of the patient ·Community, “lay” caregivers may integrate medical-centered PC service
·**A dynamic network of PC**	·May consent a better matching of time-changing needs of patients during the course of complicated diseases ·May help patients with “break-through” symptoms to gain new stability and coming back home
·**Respite care**	·It can help families to cope with cumbersome situations ·Increases the chance of patients with difficult symptoms to be managed at home

**Fig. 1:**
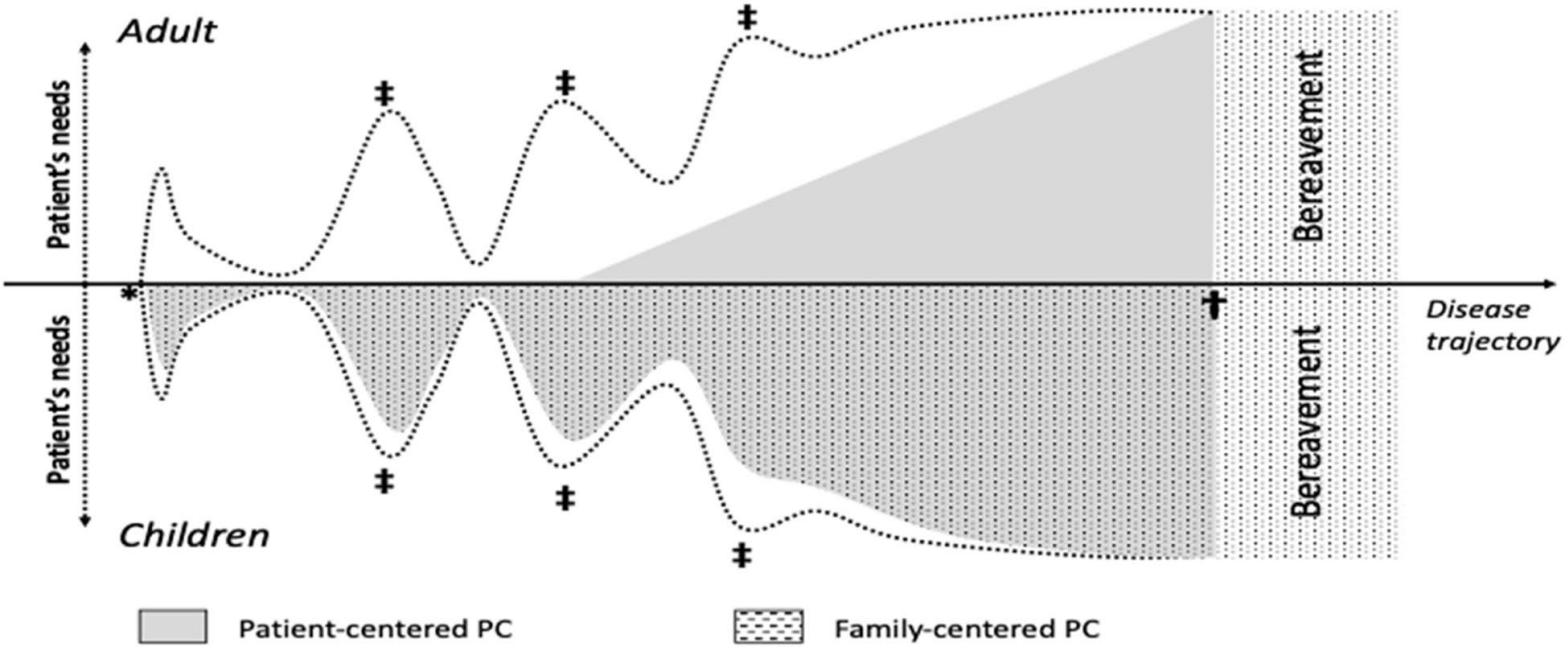
The needs of patients with life-threatening diseases change over the trajectory of disease (dotted lines; adults, above; children, below), starting at the diagnosis (*), during acute crises (‡) until death ( +). The grey area represents palliative care effectively delivered to patients; black dotted area represents the palliative care delivered to families and social background of patients

Modern oncologic treatments, including chemotherapy and radiotherapy, as well as immunotherapy, may retain a role even late in the course of oncologic disease and, in some cases, they may have a palliative role themselves. This implies an involvement of the PC team earlier than the last months, and a coordinated, simultaneous clinical activity between disease-specific clinicians and the PC team [36], see. Figure 1. This cooperative attitude may also contribute to relieve the anxiety that can be brought to a patient when he knows that he is going to be treated by the PC team, avoiding the sense of being “abandoned” by the other specialists, e.g., the oncologists, when a complete and sudden hand-over to PC team occurs. Moreover, control of pain and other signs and symptoms, including nausea, diarrhea, and neuropathy, can coexist with disease-specific treatments in earlier phases of the disease. Indeed, there is evidence that patients being offered earlier palliative care can cope better with cumbersome secondary effects of oncological treatments, thus increasing chance of success and survival rates [37]. This earlier referral to PC, which is often observed for children in PPC, can also contribute to maintain social and working competencies of patients.

Preventing social isolation can be an important part of relieving the suffering of patients approaching the last time of their life and it can help them coping with the disease, since social isolation may morally anticipate physical death. A dynamic network of palliative care services can integrate this earlier approach for the adult patients [22]. Acute palliative care units in the hospital may take care for acutely ill, palliative care patients, to obtain clinical stabilization and making it possible to discharge them at home [38]. Ideally, hospice care too could focus on stabilizing patients in the very last time of their lives, to discharge them at home and peacefully die in a comfortable place, if this is desired. Residential services should be coupled to home-based service, such as out-of-reach PC team visits or peripheral services [26]. This network should be dynamic and adapt to the changing needs of PC patients. Respite care, which has the double aim of training family caregivers and provide some emotional and time relief to families with terminally ill children, can be implemented also for adults’ cases. It may contribute to a better home care of the dying patients, partially relieving the emotional burden and lowering the general suffering associated to assisting a dying relative. As such, respite care may also reduce the need of transfers to hospital or hospice in the very last days of life. Respite care could be provided either by hospital or hospice-based staff or be part of the intervention provided at the community levels by peripheral services. In both cases, PC specialists are key players of the coordination process, aiming at providing the best relief from sufferings to patients and their families and fostering legacy in providing the best, evidence-based care [22].

Recently, animal assisted interventions (AAI), either in the forms of assisted therapies or activities, have been increasingly proposed to contribute to a better quality of life for patients within palliative care programs [39]. To be efficacious, these interventions need to be structured and included in the plan of care and delivered by specifically trained staff, either at home or in residential structures. Even allowing the favorite pet to visit the patient within a residential setting can be an alternative to a structured AAI. This approach can work particularly well with children with life-threatening illness or being treated for cancer [40]. The positive effects include a better mood, less irritation and anxiety, and a better control of somatic pain and other physical symptoms. The contact with animals may alleviate the fear of invasive procedures and it can have a measurable effect on indicators of quality of life [41]. In children, this may translate in a better coping with the hospital settings and acceptance of necessary therapies. The evidence for adults is less strict, but AAI and pet therapies can be seen as a relatively low-cost intervention that can contribute to quality of life and to a more pleasant and welcoming residential environment.

### For a global method for provision of palliative care

Taking care of either an adult approaching end-of-life (EOL) or of a child and its family requires the PC team constantly dedicating time to build a relationship and listening [42]. This allow considering all the clinical and human aspects of everyone as an essential element to approach the case and its overall management [4, 32].

PC is based on a joint workforce, including physicians, advanced practice providers, nurses, chaplains, and social workers. Some teams may include art and music therapists, pharmacists, and child-life therapists as well. These providers work with disease specialists to provide relief from suffering through communication expertise, emotional, spiritual, and psychosocial support as well as EOL care, when appropriate. Community-based palliative care may be highly effective and provide a better care than just medical-centered PC [32].

The goal of PC is to alleviate the burden of serious illness through the improvement of quality of life by addressing gaps in symptom management and communication [43]. In some patients, suffering is not just a “bodily” experience, it is not confined to physical pain or anxiety [5]; disease-related suffering may be much more complex and may include fear for the future, self-image disruption, familiar issues, potential or actual loss of working or social role, among others. A temporal dimension of suffering should be sought and validated as well, since the future projection of the actual situation may be the most cumbersome source of suffering for the patient.

The efficacious PC team is thus characterized by the firm professional and humanistic motivation to be a witness of the condition of the affected person and family, throughout their sufferings and vulnerability, and to provide the best evidence-based and progressive control of symptoms and relief of suffering, which can allow the person experiencing a low QOL to live better, and, possibly, longer.

This cumbersome work of getting to know and pointing out to the patient's life history and connecting the various specialists with respect to the clinical status and the relative perspectives, requires an empathic and active listening to the will and narrative of the patient himself (if conscious) and his family members, and to acknowledge the need for the various steps and attempts of disease specific treatment in order to understand the need of individual PC interventions [32, 44]. Only at that point, the team will be able to plan and implement the interventions to protect the person's quality of life during their disease. Some of the PC therapies, in some circumstances, can be perceived with doubt by the patient, by family members or by some other clinicians or even by some members of the team of PC itself [43]. Some interventions will imply firm professional decision-making and the implementation of shared decisions in the team needs an open discussion with the patient and with the family (re-negotiation of certain interventions or clinical initiatives, deep and painful interventions to inform the patient and family on the current clinical condition, decision about sedation process). Acknowledging and validating the autonomy of patients, whenever it is possible to elicit it, is pivotal to maintain this process linked to the basic ethical principles which underpin medical practice. Where there is a progressive preparation of the patient and the family with respect to the goals of care that, step by step, are intended to be achieved, the therapeutic path undertaken can truly be defined as a “palliative treatment path” that puts at the center of choices and actions clinics the patient, the family, and the care team itself regarding their own clarity of purpose and action.

It is as if the PC team made itself available to bring the therapeutic relationship to the level of the assisted person, towards the scientific consideration that the disease may have reached the stage of incurability and that the proposed interventions must shift to the consideration and protection of everything that can be valued (affective networks) and protected (body integrity and residual life). When time has elapsed and the patient is approaching the final moments, EOL therapies should be weighted and incremental according to the worsening of suffering.

## Conclusions

Pediatric and adult palliative care share common aims and ethical principles. Evidence, although needed, is not easy to obtain due to the particular clinical issues and high subjectivity of measurable outcomes. While adults are often referred relatively late to palliative care services during the course of a life-threatening disease, an earlier referral may consent a better control of pain and other symptoms associated with the disease and its specific treatments, and ultimately a better care of patients’ suffering. Caring of psychological and social issues for adults may contribute to lesser social isolation and better coping with the disease. A less “strict” palliative network, with either in-hospital, residential, and home-based care scenarios that dynamically interact, may add value to the quality of provided palliative care making easier to care at home for the patients. Respite care is a pediatric palliative care concept that can be implemented for adults as well and can contribute to a less need for hospital/hospice care at the end of life.

## Data Availability

Not applicable.

## Abbreviations

PC: Palliative Care
WHO: World Health Organization
APC: Adult Palliative Care
PPC: Pediatric Palliative Care
HIC: High-Income Countries
LMIC: Low-/Middle-Income Countries
QOL: Quality-Of-Life
EOL: End-Of-Life
AAI: Animal-Assisted Interventions
